# Biomedical potential of silver nanoparticles synthesized from calli cells of *Citrullus colocynthis *(L.) Schrad

**DOI:** 10.1186/1477-3155-9-43

**Published:** 2011-09-26

**Authors:** Satyavani K, Gurudeeban S, Ramanathan T, Balasubramanian T

**Affiliations:** 1 Centre of Advanced Study in Marine Biology, Faculty of Marine Sciences, Annamalai University, Parangipettai 608502, India

**Keywords:** bitter cucumber, callus extract, cell viability, HEp 2 cells

## Abstract

**Background:**

An increasingly common application is the use of silver nanoparticles for antimicrobial coatings, wound dressings, and biomedical devices. In this present investigation, we report, biomedical potential of silver nanopaticles synthesized from calli extract of *Citrullus colocynthis *on Human epidermoid larynx carcinoma (HEp -2) cell line.

**Methods:**

The callus extract react with silver nitrate solution confirmed silver nanoparticles synthesis through the steady change of greenish colour to reddish brown and characterized by using FT-IR, AFM. Toxicity on HEp 2 cell line assessed using MTT assay, caspase -3 assay, Lactate dehydrogenase leakage assay and DNA fragmentation assay.

**Results:**

The synthesized silver nanoparticles were generally found to be spherical in shape with size 31 nm by AFM. The molar concentration of the silver nanoparticles solution in our present study is 1100 nM/10 mL. The results exhibit that silver nanoparticles mediate a dose-dependent toxicity for the cell tested, and the silver nanoparticles at 500 nM decreased the viability of HEp 2 cells to 50% of the initial level. LDH activities found to be significantly elevated after 48 h of exposure in the medium containing silver nanoparticles when compared to the control and Caspase 3 activation suggested that silver nanoparticles caused cell death through apoptosis, which was further supported by cellular DNA fragmentation, showed that the silver nanoparticles treated HEp2 cells exhibited extensive double strand breaks, thereby yielding a ladder appearance (Lane 2), while the DNA of control HEp2 cells supplemented with 10% serum exhibited minimum breakage (Lane 1). This study revealed completely would eliminate the use of expensive drug for cancer treatment.

## Background

*Citrullus colocynthis *(Bitter cucumber) belongs to the family of cucurbitaceae, which are abundantly grown along the arid soils of Southeast coast of Tamil Nadu. It has a large, fleshy perennial root, which sends out slender, tough, angular, scabrid vine-like stems. The therapeutic potentials *viz*., antimicrobial [1], anti inflammatory [2], anti diabetic [3] and anti oxidant [4] effect of *Citrullus colocynthis *have reported in our laboratory. For conservation of this potent medicinal plant we have micro propagated and transplanted to the coastal region of Parangipettai.

Nanoparticles usually referred as particles with a size up to 100 nm. Nanoparticles exhibit completely new properties based on specific characteristics such as size, distribution and morphology. As specific surface area of nanoparticles is increased, their biological effectiveness can increase in surface energy [5]. Silver has long been recognized as having an inhibitory effect towards many bacterial strains and micro organisms commonly present in medical and industrial processes [6]. The most widely used and known applications of silver and silver nanoparticles are include topical ointments and creams containing silver to prevent infection of burns and open wounds [7]. Production of nanoparticles can be achieved through different methods. Chemical approaches are the most popular methods for the production of nanoparticles. However, some chemical methods cannot avoid the use of toxic chemicals in the synthesis protocol. Biological methods of nanoparticles synthesis using micro organisms [8], enzyme [9], and plant or plant extract have been suggested as possible ecofriendly alternatives to chemical and physical methods. Using plant for nanoparticles can be advantageous over other biological processes by eliminating the elaborate process of maintaining cell culture [10]. If biological synthesis of nanoparticles can compete with chemical methods, there is a need to achieve faster synthesis rates. The exact mechanism of silver nanoparticles synthesis by plant extracts is not yet fully understood. Only participation of phenolics, proteins and reducing agents in their synthesis has been speculated. Recently nano-encapsulated therapeutic agents such as antineoplastic drugs had been used to selectively targeting anti tumor agents and obtaining higher drug concentration at the tumour site [11]. Nanotechnology could be very helpful in regenerating the injured nerves. For biological and clinical applications, the ability to control and manipulate the accumulation of nanoparticles for an extended period of time inside a cell can lead to improvements in diagnostic sensitivity and therapeutic efficiency. This when revealed completely would eliminate the use of expensive drugs for cancer treatment [12]. The callus and leaf extract of *Citrullus colocynthis *reported moderate antimicrobial activity against biofilm forming bacteria [13] and harmful human pathogens [14]. Therefore the present study, we evaluated, biomedical potential of silver nanopaticles synthesized from calli extract of *Citrullus colocynthis *on Human epidermoid larynx carcinoma (HEp -2) cell line.

## Results and Discussion

**Figure 1:**
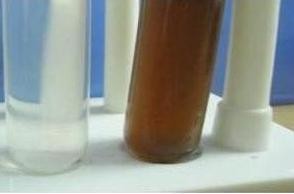
**1 mM silver nitrate solution without callus extract and silver nanoparticles with reddish brown colour**. 1 mM silver nitrate solution without callus can be seen in A and silver nanoparticles with reddish brown colour can be seen in B.

**Figure 2:**
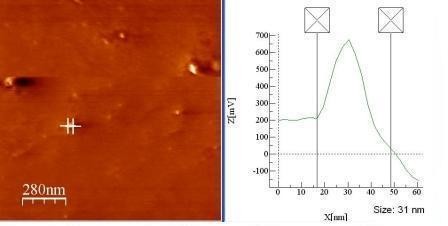
**AFM**. Tapping mode AFM (VEeco diNanoscope 3D AFM) image showed spherical shaped silver nanoparticles with size range 31 nm.

The cumulative work on plant tissue culture revealed the maximum number of calli induction was achieved from stem explants of *C. colocynthis *on MS medium enriched with 0.5 mg L-1 IAA, 2, 4-D and 1 ppm of 6-BA which yielded morphogenic compact hard greenish white calli at a frequency of 80%. The appearance of brown colour in the reaction mixture indicates the synthesis of silver nanoparticles form stem derived callus extract with 1 mM silver nitrate solution (Figure 1). Our findings showed resemblance to the results already reported by in the case of callus extract of *Carcia papaya *[15], leaf extract of *Capsicum annum *[16] and in case of extract of *Aloe Vera *[17]. The shape of the SNP synthesized by stem derived callus extract was spherical and was found to be in the range 31 nm by AFM (Figure 2).

**Figure 3:**
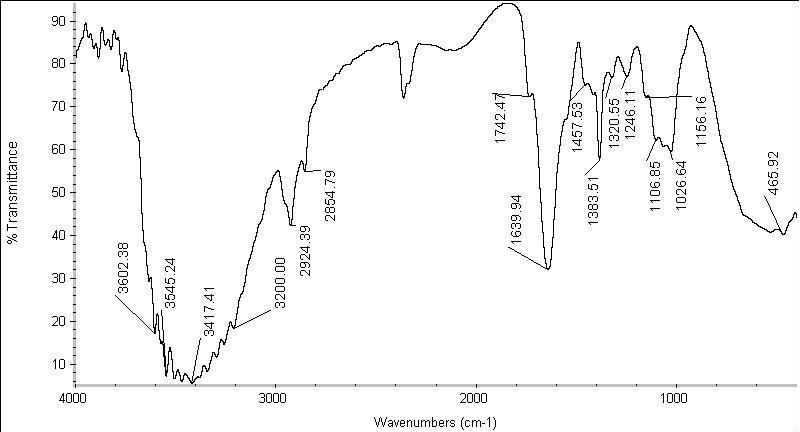
**FT-IR**. FT-IR images identified silver nanoparticles associated biomolecules. It represents compounds attached with silver nanoparticles could be polyphenols with aromatic ring and bound amide region in the peaks ranging from 1000-4000 cm^-1^.

Number of absorption spectrum of the nanoparticles obtained in the present study as shown in (Figure 3). Among them, the absorption peak at 1020 cm^-1 ^can be assigned a absorption peaks of C-O-C- or -C-O-, also the peak at 1020-1091 cm^-1 ^corresponds to C-N stretching vibrations of aliphatic amines or to alcohols or phenols representing the presence of polyphenols [18]. The absorbance peak at 1265 and 1384 - 1460 cm^-1 ^correspond to the amide III and II group respectively. The peak at 1624 cm^-1 ^is associated with stretch vibration of -C = C-and is assigned to the amide 1 bonds of proteins. The absorption at about 1384 cm^-1 ^is notably enhanced indicating residual amount of NO_3 _in the solution [19]. The peak at 1539 cm^-1 ^may be assigned to symmetric stretching vibrations of -COO- (carboxyl ate ion) groups of amino acid residues with free carboxyl ate groups in the protein [20]. The peak at 3427 cm^-1 ^indicates polyphenolic OH group along with the peak of 882 cm^-1 ^which represents the aromatic ring C-H vibrations, indicate the involvement of free catechin [21]. This suggests the attachment of some polyphenolic components on to silver nanoparticles. This means the polyphenols attached to silver nano particles may have atleast one aromatic ring. The peaks at 1000-1200 cm^-1 ^indicate C-O single bond and peaks at 1620-1636 cm^-1 ^represent carbonyl groups (C = O) from polyphenols such as catechin gallate, epicatechin gallate and theaflavin [22]. Result suggests that molecules attached with silver nanoparticles have free and bound amide group. These amide groups may also be in the aromatic rings. This concludes that the compounds attached with silver nanoparticles could be polyphenols with aromatic ring and bound amide region. In our results showed that the average number of atoms per nanoparticles are N = 914047.97. The molar concentration of the silver nanoparticles solution in our present study is 1100 nM/10 mL.

### Toxicity study

**Figure 4:**
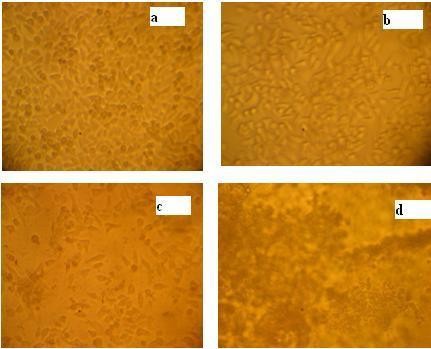
**Dose dependent Cytotoxicity assay**. Dose dependent cytotoxicity effect of SNp over cell viability (a) Normal Hep-2 cells (b) Low toxicity 15.5 μg/ml (c) Minimum toxicity 500 μg/ml (d) high toxicity 1000 μg/ml.

**Figure 5:**
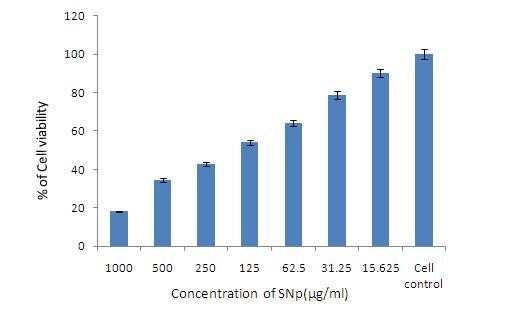
**MTT assay**. Cytotoxicity of different concentration (15.25 -1000 μg/ml) of silver nanoparticles measured by MTT assay on Hep2 cell line.

The nanoparticles synthesized using the plant system have applications in the field of medicines, cancer treatment, drug delivery, commercial appliances and sensors. The *in vitro *cytotoxicity effects of silver nanoparticles were screened against cancer cell lines and viability of tumor cells was confirmed using MTT assay. The silver nanoparticles were able to reduce viability of the HEp -2 cells in a dose-dependent manner as shown in (Figure 4 &5). After five hours of treatment, the silver nanoparticles at concentration of 500 nM decreased the viability of HEp 2 cells to 50% of the initial level, and this was chosen as the IC_50_. Longer exposures resulted in additional toxicity to the cells. These results demonstrate that silver nanoparticles mediate a concentration and time dependent increase in toxicity. Silver nanoparticles had important anti angiogenic properties [23], so are attractive for study of their potential antitumor effects. The toxicity of nanosilver on oestoblast cancer cell lines results demonstrate a concentration-dependent toxicity with 3.42 *μ*g/ml of IC_50 _suggest that the product is more toxic to cancerous cell comparing to other heavy metal ions [24]. Therefore our tissue culture derived silver nanoparticles of *Citrullus colocynthis *serve as antitumor agents by decreasing progressive development of tumor cells.

According to the levels of lactate dehydrogenase (LDH) released into the medium of control and synthesized silver nanoparticles treated (20, 40, 60, 80 and 100 μg/ml) HEp2 cells are presented in Table 1. From this table, it was observed that LDH activities found to be significantly elevated after 48 h of exposure in the medium containing silver nanoparticles when compared to the control.

**Table 1:** Cell viability and LDH Leakage in control and SNp, treated HEp2 cells after 48 h of exposure

Concentration (μg/ml)		Percentage of inhibition	LDH activity (μmol of NADH/per well/min.)
	Control	0	0.10 ± 0.004
	DMSO 1% (v/v)	0	0.12 ± 0.005
*SNp*	20 (μg/ml)	0	0.14 ± 0.006*
	40 (μg/ml)	21.98 ± 1.47*	0.20 ± 0.01*
	60 (μg/ml)	50.14 ± 1.24*	0.38 ± 0.02*
	80 (μg/ml)	67.60 ± 1.42*	0.46 ± 0.02*
	100 (μg/ml)	91.84 ± 1.28*	0.57 ± 0.02*

Each values represents mean + SD of 3 replicates * P < 0.001 Vs Control

**Figure 6:**
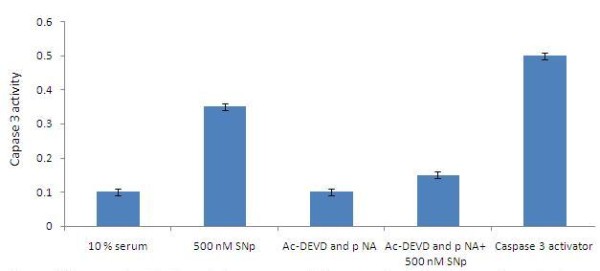
**Capase 3 assay**. Capase 3 activation of silver nanoparticles caused cell death through apopotosis p < 0.05 vs control, data Mean standard deviation from 3 replicates (n = 3; p < 0.01).

**Figure 7:**
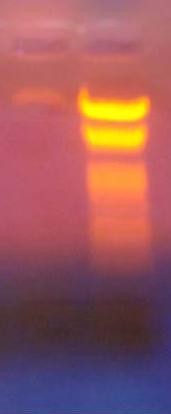
**DNA fragmentation assay**. DNA fragmentation assay lane 1 (10% serum) and lane 2 (treated with SNp).

Also, the cellular metabolic activity affected by the silver nanoparticles, the possibility of apoptosis induction by the nanoparticles was assessed, especially at the IC_50_. Levels of caspase 3, a molecule which plays a key role in the apoptotic pathway of cells, were increased following the treatment with silver nanoparticles. The cell lysates obtained from HEp2 cells treated with silver nanoparticles at 500 nM concentrations for six hours was used for this assay. Caspase 3 activation suggested that silver nanoparticles caused cell death through apoptosis, which was further supported by cellular DNA fragmentation. DNA ladders of the corresponding treated samples confirmed apoptosis (Figure 6) and showed that the silver nanoparticles treated HEp2 cells exhibited extensive double strand breaks, thereby yielding a ladder appearance (Lane 2), while the DNA of control HEp2 cells supplemented with 10% serum exhibited minimum breakage (Lane 1) (Figure 7).

However, when compared as a function of the Ag^+ ^concentration, toxicity of AgNP appeared to be much higher than that of AgNO_3 _[25]. The cytotoxic effects of silver are the result of active physicochemical interaction of silver atoms with the functional groups of intracellular proteins, as well as with the nitrogen bases and phosphate groups in DNA [26]. Regular green tea and decaffeinated green tea exhibit dose-dependent inhibitory activity in (H1299 cell line) human lung carcinoma cell line. Also the apoptosis mechanism is induced in the presence of polyphenols concentrations were less [27].

This may be due to their inhibitory activities in several signaling cascades responsible for the development and pathogenesis of the disease which are as yet not understood. Taken together, our data suggest that silver nanoparticles can induce cytotoxic effects on HEp -2 cells, inhibiting tumor succession and thereby effectively controlling disease progression without toxicity to normal cells and these agents an effective alternative in tumor and angiogenesis-related diseases.

## Conclusion

In conclusions, plant based sliver nanoparticles possess considerable anticancer effect compared with commercial nanosilver. The reduction of the metal ions through the callus extracts leading to the formation of silver nanoparticles of fairly well defined dimensions. Use of AgNPs should emerge as one of the novel approaches in cancer therapy and, when the molecular mechanism of targeting is better understood, the applications of AgNPs are likely to expand further [28].

## Materials and Methods

### Plant material and preparation of the extract

Fresh *Citrullus colocynthis *leaves were collected from the Southeast coast of Parangipettai (Tamil Nadu) India. The specimen was certified by Botanical Survey of India (BSI) Coimbatore, and documented in the Herbaria of C.A.S. in Marine Biology, Annamalai University, India, during 2010. The experimental chemicals were purchased from Sigma Chemicals (Mumbai).

### Sample preparation for synthesis of Silver Nanoparticles

One month old compact, hard greenish white callus derived from stem explants was used to obtain the callus extract in our lab [29]. The callus was washed twice with sterile distilled water to remove medium components before grinding. Approximate 20 g of callus was grinded in 100 ml of sterile distilled water in mortar and pestle. The resulting extract was filtered through filter paper (What man No.1) and used for the synthesis of silver nanoparticles. 10 ml suspension of callus culture was added to 90 ml aqueous solution of silver nitrate (1 mM) solution separately

for reduction in to Ag+ ions and incubated at room temperature (35°C) for about 24 hours. The primary detection of synthesized silver nanoparticles was carried out in the reaction mixture by observing the colour change of the medium from greenish to dark brown. The silver nanoparticles were isolated and concentrated by repeated (4-5 times) centrifugation of the reaction mixture at 10, 000 g for 10 min. The supernatant was replaced by distilled each time and suspension stored as lyophilized powder for the optical measurements.

### Atomic Force Microscope

Purified SNP in suspension was also characterized their morphology using a VEeco diNanoscope 3D AFM (Atomic Force Microscope). A small volume of sample was spread on a well-cleaned glass cover slip surface mounted on the AFM stub, and was dried with nitrogen flow at room temperature. Images were obtained in tapping mode using a silicon probe cantilever of 125 μm length, resonance frequency 209-286 kHz, spring constant 20-80 nm^-1 ^minimum of five images for each sample were obtained with AFM and analyzed to ensure reproducible results.

### Fourier Transform Infra Red Spectroscope

To identify Silver nanoparticles associated biomolecules, the Fourier transform infra red spectra of washed and purified Silver nanoparticles powder were recorded on the Nicolet Avatar 660 FT-IR Spectroscopy (Nicolet, USA) using KBr pellets. To obtain good signal to noise ratio, 256 scans of Silver nanoparticles were taken in the range of 400-4000 cm-1 and the resolution was kept as 4 cm^-1^

### Determination of Nanoparticles concentration

Accurate determination of the size and concentration of nanoparticles is essential for biomedical application of nanoparticles [30]. The concentration of nanoparticles to be administered at an nM level of determination by Marquis method [31].

### Toxicity Study of SNp on Human Epidermoid Larynx Carcinoma (HE_P _-2) Cell Line

#### Cell Culture

HEp-2 cell line was purchased from National Cell Centre, Pune (India). Cancerous cells were seeded in flask with MEM medium with 2-10% Fetal Calf Serum (FCS) and incubated at 37°C in a 5% CO_2 _atmosphere. After 48 h incubation period, the attached cells were trypsinated for 3- 5 mints and centrifuged at 1, 400 rpm for 5 mints. The cells counted and distributed in 24 well micro titer plates with 10, 000 cells in each well and incubated 48 hrs at 37°C in a 5% CO_2 _atmosphere for the attachment of cells to bottom of the wells.

#### Cell Treatment with silver nanoparticles

The amount of different concentrations of stabilized silver nanoparticles was added to each well in duplicates. The different silver nanoparticles concentrations (15, 30, 62, 125, 250, 500, 1000 μg/ml) were inoculated in to grown cell (1 × 10 ^4 ^cells/well) and the cell population was determined by optical microscopy at 24 and 48 hrs.

#### MTT assay

Cell viability was evaluated by MTT colorimetric technique [30]. 200 μl of the yellow tetrazolium (MTT (3-(4, 5-dimethylthiazol-2)-2, 5 diphenyl tetrazolium bromide) without phenol red, are yellowish in color (Sigma) solution (5 mg/mL in PBS) was added to each well. The plates were incubated for 3-4 h at 37°C, for reduction of MTT by metabolically active cells, in part by the action of dehydrogenase enzymes, to generate reducing equivalents such as NADH and NADPH. The resulting intracellular purple formazan solubilized the MTT crystals by adding and quantified by spectrophotometric mean and then the supernatants were removed. For solubilization of MTT crystals, 100 μl DMSO was added to the wells. The plates were placed on a shaker for 15 mints for complete solubilization of crystals and then the optical density of each well was determined. The quantity of formazan product was measured by the amount of 545 nm absorbance is directly proportional to the number of living cells in culture. The relative cell viability (%) related to control wells containing cell culture medium without nanoparticles as a vechicle was calculated by [A]_test_/[A]_control_×100. Where [A]_test _is the absorbance of the test sample and [A]_control _is the absorbance of control sample

#### Lactate Dehydrogenase (LDH) leakage assay

Intracellular lactate dehydrogenase (LDH) leakage, a well known indicator of cell membrane integrity and cell viability was performed by the method of Borna *et al.*, (2009) [31]. 100 ∞ l of silver nanoparticles was added to a 1 ml cuvette containing 0.9 ml of a reaction mixture to yield a final concentration of 1 mM pyruvate, 0.15 mM NADH and 10^4 ^mM disodium hydrogen phosphate. After mixing thoroughly, the absorbance of the solution was measured at 340 nm for 45 seconds. LDH activity was expressed as moles of NADH used per minute per well.

#### Caspase 3 assay

Caspase-3 is an intracellular cysteine protease that exists as a proenzyme, becoming activated during the cascade of events associated with apoptosis. Caspase-3 cleaves a variety of cellular molecules that contain the amino acid motif DEVD such as poly ADP-ribose polymerase (PARP), the 70 kD protein of the U1-ribonucleoprotein and a subunit of the DNA dependent protein kinase [32]. The presence of caspase-3 in cells of different lineages suggests that caspase-3 is a key enzyme required for the execution of apoptosis [33]. The cells were lysed with the lysis buffer provided in the caspase 3 assay kit (Sigma, USA) and kept on ice for 15-20 minutes. The assay is based on the hydrolysis of the peptide substrate, Ac-DEVD-pNA, by caspase 3, resulting in the release of Ac-DEVD and p nitroaniline (pNA) which absorbs light significantly at 450 nm. Briefly, for 1 mL of the reaction mixture, 10 mL of the cell lysate from treated samples was added along with 980 mL of assay buffer, followed by addition of 10 mL of 20 mM caspase 3 colorimetric substrate (Ac-DEVD pNA). The cell lysates of the SNp-treated Hep-2 cells were then incubated at 37°C with the caspase 3 substrate for two hours and the absorbance was read at 450 nm in a double-beam UV- spectrophotometer (Shimadzu, Japan). The assay was also performed with noninduced cells and in the presence of caspase 3 inhibitor for a comparative analysis.

#### DNA fragmentation assay

DNA fragmentation has long been used to distinguish apoptosis from necrosis, and is among the most reliable methods for detection of apoptotic cells. When DNA strands are cleaved or nicked by nucleases, 3'-hydroxyl ends are exposed. 1 × 10^6 ^cells were lysed in 250 μL cell lysis buffer containing 50 mM Tris HCl, pH 8.0, 10 mM ethylenediaminetetraacetic acid, 0.1 M NaCl, and 0.5% sodium dodecyl sulfate. The lysate was incubated with 0.5 mg/mL RNase A at 37°C for one hour, and then with 0.2 mg/mL proteinase K at 50°C overnight. Phenol extraction of this mixture was carried out, and DNA in the aqueous phase was precipitated by 25 μL (1/10 volume) of 7.5 M ammonium acetate and 250 μL (1/1 volume) isopropanol. DNA electrophoresis was performed in a 1% agarose gel containing 1 μg/mL ethidium bromide at 70 V, and the DNA fragments were visualized by exposing the gel to ultraviolet light, followed by photography.

#### Statistical analysis

All experiments were done in duplicate and then values were expressed as mean ± standard deviation (SD). Statistical significance (5%) was evaluated by one-way analysis of variance (ANOVA) followed by Student's t-test (p < 0.05, SPSS 11 version).

## Competing interests

A patent application will be filed with the content of this article, through the Annamalai University. The authors declare that they have no competing interests.

## Authors' contributions

All authors read and approved the final manuscript.

KS and SG developed the concept and designed experiments. TR was research guide of this experimental study. SG and KS performed plant collection, micropropagation, nanoparticles synthesis, characterization and cell line studies. TR & TB provided chemicals, Instrumental studies and advised on experimental part.
